# Human Blood‐Brain Tumor Barrier on a Chip to Investigate Personalized Treatment for Glioblastoma Patients

**DOI:** 10.1002/smll.202506712

**Published:** 2026-06-27

**Authors:** Minsu Ryoo, Gaeun Lee, Jinwoo Jung, Sujin Cho, Sharon Jeeho Ham, Nayeong Kang, Hyeongjin Ahn, Yu Jin Kim, JeongMin Sim, Jeongman Park, Juwon Kim, Sohyun Hwang, Jihwan Yoo, Youn‐Jung Kang, Jaejoon Lim, Jungho Ahn, Song Ih Ahn

**Affiliations:** ^1^ Department of Mechanical Engineering Korea Advanced Institute of Science and Technology Daejeon Republic of Korea; ^2^ Department of MetaBioHealth Sungkyunkwan University Suwon Republic of Korea; ^3^ School of Mechanical Engineering Pusan National University Busan Republic of Korea; ^4^ Department of Biophysics Sungkyunkwan University Suwon Republic of Korea; ^5^ Department of Biomedical Science School of Life Science CHA University Seongnam Republic of Korea; ^6^ Department of Pathology Bundang CHA Medical Center CHA University Seongnam Republic of Korea; ^7^ Department of Neurosurgery Brain Tumor Center Gangnam Severance hospital Seoul Republic of Korea; ^8^ Department of Biochemistry Research Institute for Basic Medical Science School of Medicine CHA University Seongnam Republic of Korea; ^9^ Department of Neurosurgery Bundang CHA Medical Center CHA University Seongnam Republic of Korea

**Keywords:** blood‐brain tumor barrier, drug screening, glioblastoma, organ‐on‐a‐chip, personalized models

## Abstract

The inherent characteristics of glioblastoma (GBM), including tumoral heterogeneity and invasive capacity, combined with the presence of the blood‐brain tumor barrier (BBTB), present challenges in developing effective treatment for GBM. Especially, the margins of GBM, where GBM cells infiltrate normal brain tissue, exhibit high resistance to therapies. Despite the difficulties in controlling tumor progression within this region, the GBM margin remains a critical area to be studied. Here we report a microengineered model that mimics the BBTB within the GBM margin, incorporating a 3D network of normal astrocytes and GBM cells isolated from patients newly diagnosed with GBM. The interaction between GBM cells and stromal cells results in increased vascular permeability, reactive gliosis, alterations in astrocyte behavior and immune responses to foster tumor invasiveness and progression. We compare patient‐specific tumor responses to conventional chemotherapy and immune polarization in our BBTB on a chip model with clinical outcomes, demonstrating the capability of the model to predict personalized drug responses. Our BBTB model may serve as a personalized tool to examine the interactions between tumors and normal brain tissue, ultimately facilitating the screening of personalized medicine for GBM treatment.

## Graphical abstract

A microfluidic model of the glioblastoma (GBM) margin was developed to mimic patient‐specific blood‐brain tumor barrier (BBTB) function and drug response. The platform revealed inter‐patient variability in barrier integrity and drug sensitivity, offering a tool for personalized therapy evaluation in GBM.

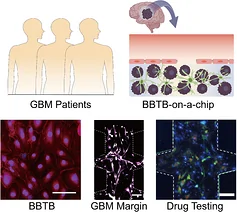

## 1 Introduction

Glioblastoma (GBM) is the most prevalent and aggressive type of brain cancer, with a median survival of less than 15 months despite multimodal therapy [1, 2]. The poor prognosis of GBM is primarily due to its intrinsic heterogeneity across patients and diffusively infiltrative growth pattern, which hinders complete surgical resection and facilitates recurrence [3, 4]. A particularly challenging region is the GBM margin, where invasive tumor cells infiltrate normal brain parenchyma [5, 6]. In this region, GBM cells interact dynamically with resident glial cells [7] and are shielded by the blood‐brain tumor barrier (BBTB), a pathological extension of the blood–brain barrier (BBB), that retains a relatively intact vascular barrier [8]. These features not only restrict drug delivery but also contribute to therapy resistance and tumor progression. Therefore, understanding the cellular and barrier dynamics at the tumor margin is critical for improving therapeutic outcomes in GBM.

The heterogeneity of GBM, both within tumors and across patients, contributes significantly to the inconsistent therapeutic responses, even among patients with favorable prognostic markers such as methylated O(6)‐Methylguanine‐DNA methyltransferase (MGMT) [9]. Although patient's genomic profiling provides important diagnostic and prognostic insights, it does not fully capture the functional differences in tumor biology, particularly in relation to drug permeability and therapeutic response. These limitations highlight the need for patient‐specific models that can recapitulate the microenvironment and vascular barrier characteristics of the GBM margin, and rapidly evaluate individual therapeutic responses [10].

Microphysiological systems have emerged as promising platforms for personalized medicine screening, by combining patient‐derived cells with physiologically relevant microenvironments [11]. This technology has adopted diverse strategies to replicate the GBM features, including the integration of GBM spheroids [12, 13], the simulation of hypoxic conditions [11, 14, 15], or the use of patient‐derived cells [11, 16, 17]. However, few models have successfully recapitulated the integrated features of the BBTB within a patient‐specific GBM margin [18].

**FIGURE 1:**
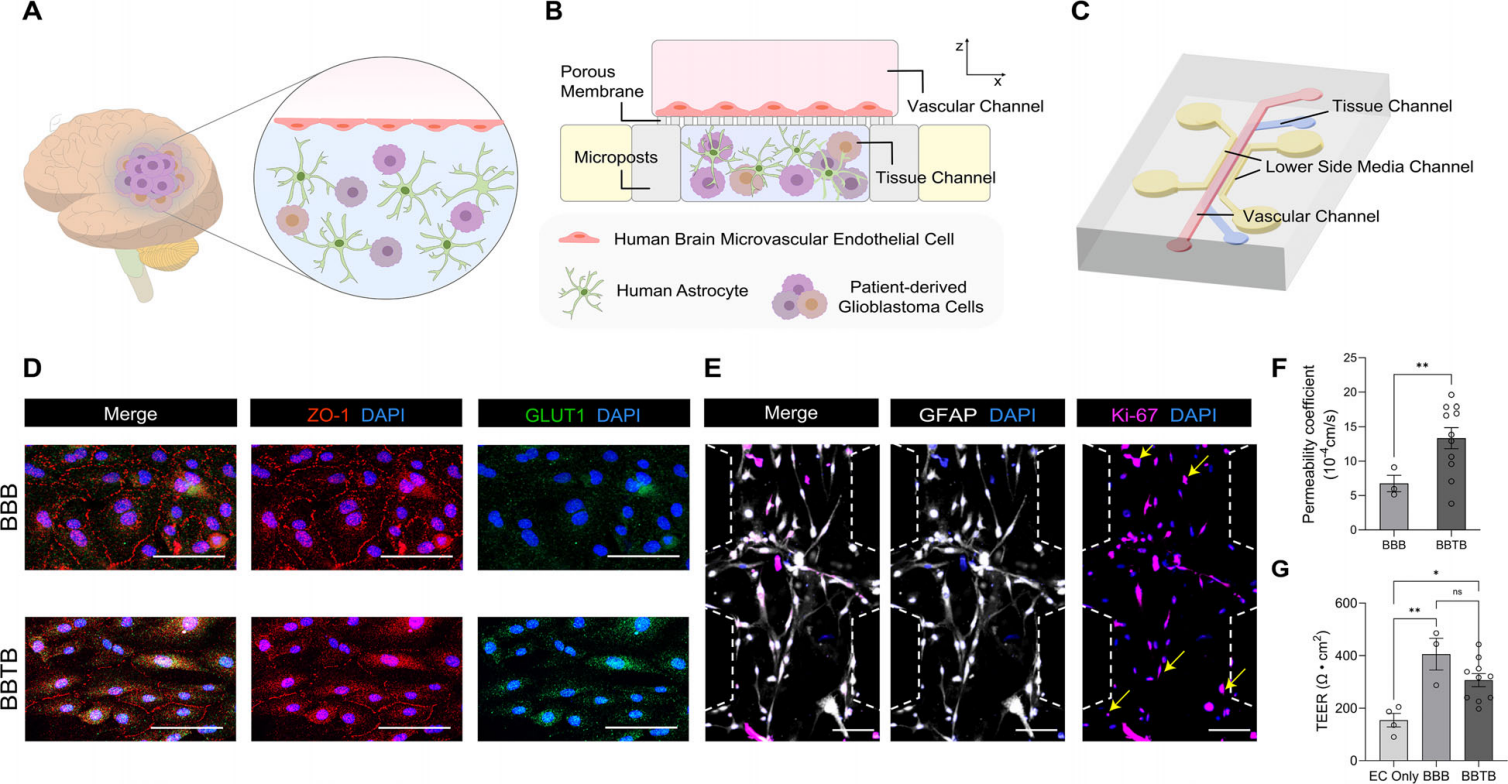
Microengineered human blood–brain‐tumor barrier (BBTB) model. (A) Schematic representation of the BBTB within the glioblastoma (GBM) margin. (B) Cross‐sectional view of our BBTB model consisting of the human brain endothelial barrier in vascular channel (upper channel) and a 3D network of astrocytes and patient‐derived GBM cells in tissue channel (lower channel). The two microfluidic layers are separated by a porous membrane, and the tissue channel and lower side media channel are separated by an array of microposts. (C) The microfluidic chip with the vascular channel (red), the tissue channel (blue), and the lower side media channels (yellow). (D) Human brain microvascular endothelial cell (HBMEC) monolayers formed in the vascular channel of the healthy blood‐brain barrier (BBB) and BBTB models (ZO‐1, red; GLUT1, green; DAPI, blue) (All scale bars = 100 µm). (E) Tissue layer showing co‐cultured GBM and astrocytes (GFAP, white; Ki‐67, magenta; DAPI, blue) (All scale bars = 100 µm). Yellow arrows indicate cells with visibly distinct Ki‐67 immunofluorescence signals. (F) Permeability coefficients of BBB and BBTB (n = 3 for BBB, n = 11 for BBTB, **p < 0.01 by student's t‐test, error bars represent the standard error of the mean (SEM)). (G) TEER measurements for endothelial cells only (EC Only), BBB, and BBTB conditions, reflecting the integrity of the barrier (n = 4 for EC only, n = 3 for BBB, and n = 10 for BBTB, *p < 0.05 and **p < 0.01 by one‐way ANOVA with Tukey's post hoc test, error bars represent the standard error of the mean (SEM)).

In this study, we present a microphysiological system that recapitulates the 3D microenvironment of the BBTB within the GBM margin (Figure 1A; Figure S1). Our model is engineered to reconstruct the brain vascular barrier and perivascular environment of GBM within a double‐layered microfluidic device, enabling spatially organized co‐culture of patient‐derived GBM cells and astrocytes that reflects the infiltrative margin architecture [19]. GBM cells were isolated from three GBM patients with MGMT promoter methylation, a marker predictive of response to chemotherapy with alkylating agents like Temozolomide (TMZ) [20, 21, 22, 23]. Despite sharing the same MGMT promoter methylation status, the patient‐derived models exhibited apparent differences in vascular phenotypes, drug responsiveness, and immune signatures, reflecting inter‐patient heterogeneity. Our platform also enables the incorporation of pericytes and microglia, to more comprehensively reconstruct the BBTB microenvironment and assess therapeutic response and immune polarization in a patient‐specific manner. Altogether, this model provides a translational tool to functionally evaluating individualized BBTB characteristics and informing personalized therapeutic strategies for GBM.

## 2 Materials and Methods

### 2.1 Fabrication of the Microfluidic Device

The microengineered device was fabricated using polydimethylsiloxane (PDMS; Sylgard 184, Dow Corning, Midland, MI, USA) via soft lithography. The PDMS elastomer base was mixed with a curing agent at a 10:1 (w/w) ratio. After degassing to remove air bubbles, the mixture was poured over silicon wafers patterned with SU‐8 photoresist (Microfit, Hanam‐si, Gyeonggi‐do, Republic of Korea) and baked at over 80°C for 1 h to allow curing. Subsequently, the solution was poured onto the lower‐layer wafer, and spin‐coating was applied to achieve a lower channel height of 250 µm. This lower layer was then cured on a hot plate at 130°C for 18 min. Prior to assembly, both the upper and lower layers were cut, and media reservoir holes in the upper layer were punched using a 4 mm biopsy punch (KAI Medical, Seki, Gifu, Japan), while other inlet and outlet ports were created using a 1 mm punch. Both sides of the polycarbonate membrane with 8 µm pores (Sterlitech Corp, Kent, WA, USA) were plasma treated using a vacuum plasma system (Femto Science, Hwaseong‐si, Gyeonggi‐do, Republic of Korea) for 1 min. The plasma‐treated membranes were then cut into 10 mm × 3 mm pieces and immersed in a 5% solution of 3‐aminopropyltriethoxysilane (APTES; Sigma‐Aldrich, St. Louis, MO, USA) at 80°C for 20 min. Finally, the membrane was sandwiched between the upper and lower PDMS layers and bonded using the same vacuum plasma system. The assembled device and microchannels were sterilized with 70% ethanol, followed by incubation in a dry oven at 80°C for over 2.5 days to restore the hydrophobicity of the PDMS surface.

### 2.2 GBM Cell Isolation

The patient tissues were collected from CHA Bundang Medical Center, Republic of Korea, under Institutional Review Board approval (No. CHAMC2021‐01‐024‐001); all patients provided informed consent. Tissue sampling was standardized across all patients. Both 5‐ALA fluorescence guidance and neuronavigation were used to identify and collect tissue specifically from peripheral tumor regions exhibiting distinct 5‐ALA fluorescence, corresponding to vascularized and actively infiltrating margin areas. Tissues were surgically obtained and stored at −80°C. The patient sample was diagnosed by a neuropathologist and also underwent next‐generation sequencing using the Oncomine Comprehensive Assay.

Primary GBM cells were acquired from histological grades 4 GBM tissue samples. Tissues were mechanically sectioned into small pieces and enzymatically dissociated using collagenase D (Roche Diagnostics, Basel, Switzerland) and DNase I (Roche Diagnostics) at 37°C for 30 min. The cells were washed with phosphate‐buffered saline (PBS; Thermo Fisher Scientific, Waltham, MA, USA) and enumerated using a hemocytometer (Thermo Fisher Scientific). Then, cells, at a density of 1E6 cells/mL, were cultivated in Dulbecco's modified eagle medium (DMEM) supplemented with 12.5% FBS, 1% (v/v) penicillin/streptomycin, 50 ng/mL epidermal growth factor (EGF; Thermo Fisher Scientific), and 50 ng/µL fibroblast growth factor (FGF; PeproTech, Cranbury, NJ, USA) at 37°C and in a 5% CO2 atmosphere. All media and reagents were lipopolysaccharide (LPS)‐free. FBS was heat‐inactivated and contained <5 EU/mL of endotoxin.

### 2.3 Molecular Subtype Signature Analysis

Transcriptomic molecular subtype signatures (Proneural, Classical, Mesenchymal) were analyzed using Python (v3.10). Raw count matrices were imported using Pandas, and genes were log‐normalized prior to analysis. For each sample, expression levels of subtype‐defining gene signatures were extracted, z‐score transformed, and visualized as a subtype heatmap [24]. Subtype designation was determined based on the dominant signature pattern for each patient. Heatmaps were generated using seaborn and matplotlib.

### 2.4 Microglia Analysis

Microglial polarization was assessed using transcriptomic expression of established M1 (CD86, IRF5, IL1B, TNF) and M2 (CD206/MRC1, ARG1, IL10) marker genes. Normalized expression values were averaged within each gene set to generate M1 and M2 signature scores, and M1/M2 ratios were calculated to compare microglial polarization across patient‐derived models.

### 2.5 Next‐Generation Sequencing (Targeted DNA Panel)

We analyzed FFPE GBM tumor tissues from four patients using targeted NGS. Genomic DNA was extracted using the RecoverAll Multi‐Sample RNA/DNA Isolation Workflow (Invitrogen, Carlsbad, CA, USA). Libraries were prepared using the Oncomine Comprehensive Assay Plus (OCAplus, Thermo Fisher Scientific), covering 501 cancer‐associated genes and including MSI and TMB modules. Sequencing was performed on the Ion Torrent S5 XL system. Reads were aligned to hg19 using Torrent Suite v5.20. Variant calling and annotation were performed with Ion Reporter v5.20. MSI‐high was defined as ≥19 and high TMB as >10 mutations/Mb.

### 2.6 Cell Culture

Human Brain Microvascular Endothelial Cells (HBMEC; Innoprot, Bizkaia, Spain) at passages 3–5 were cultured in endothelial cell medium (Sciencell, San Diego, CA, USA) on flasks coated with 50 µg/mL fibronectin (Sigma‐Aldrich). Human Astrocytes (HA; Sciencell) at passages 3–5 were cultured on flasks coated with 1 mg/mL poly‐l‐lysine (PLL; Sigma‐Aldrich) and maintained in astrocyte medium (Sciencell). GBM cells from patients at passage 3–5 were grown in Dulbecco's Modified Eagle Medium F12 (DMEM/F12, Gibco, NY, USA) with the addition of 50 ng/mL Recombinant Human Epidermal Growth Factor (EGF, Peprotech, Cedarbrook Dr, USA) and 50 ng/mL Recombinant Human Fibroblast Growth Factor‐10 (FGF‐10 Peprotech) on flasks (Figure S2). Human Brain Vascular Pericytes (HBVP; Sciencell) at passages 3–5 were cultured on flasks coated with 1 mg/mL poly‐l‐lysine (PLL; Sigma‐Aldrich) and maintained in pericyte medium (Sciencell). Microglia cell line HMO6 was gifted by Prof. S. Park of Sungkyunkwan University, which was invaluable for this study. Microglia were cultured on flasks coated with 1 mg/mL poly‐l‐lysine (PLL; Sigma‐Aldrich) and grown in microglia medium (MM) (Dulbecco's Modified Eagle Medium—high glucose F12 (D6429, Gibco)).

### 2.7 Construction of HBMEC–astrocyte–GBM Chip System

HAs and GBM cells were labeled with CellTracker (CellTracker Red CMTPX Dye, Thermo Scientific; CellTracker Green CMFDA Dye, Thermo Scientific) and then incubated at 37°C for 30 min before seeding into the chips. HAs and GBM cells, with a density of 2E6 cells/mL, were enclosed in a precursor solution of growth factor reduced Matrigel (Matrigel; Corning, NY, USA) at a concentration of 5 mg/mL and loaded in the lower center channel. The cell loaded Matrigel was incubated for 1 h at 37°C to allow gelation. After 1 h, mixed cell culture medium (1:1 of astrocyte medium: endothelial cell medium), was introduced into the lower side channel to provide nourishment to the embedded HAs and GBM cells, preventing Matrigel shrinkage. The chip was subsequently incubated at 37°C for 24 h to promote the proliferation of HAs and GBM cells prior to the introduction of HBMECs. Before seeding HBMECs onto the membrane in the upper channel, the channel was coated with 50 µg/mL fibronectin and incubated for 30 min at 37°C to enhance the attachment of cells onto the surface of channels. Afterward, HBMECs were seeded into the upper channel at a density of 7E7 cells/mL. After 1 h, the mixed cell culture medium was replenished in the upper channel to remove unattached cells and supply nutrients to HBMECs. The chips were incubated at 37°C with 5% CO_2_ for 3 days, with the cell medium being refreshed twice a day.

### 2.8 Construction of Penta‐Culture Model with Microglia and Pericytes

First, the lower center channel was coated with 50 µg/mL fibronectin for 30 min at 37°C to support cell adhesion. HBVPs were then seeded into the lower center channel at a density of 1E7 cells/mL, and the chip was immediately flipped upside down to promote attachment. After 1 h, cell culture medium was refreshed in the upper channel to remove unattached cells and supply nutrients to HBVPs. After 6 h, the chip was returned to its upright position, and HAs, microglia and GBM cells, with a density of 2E6 cells/mL for each cell type, were suspended in a precursor solution of growth factor reduced Matrigel (5 mg/mL; Corning) and loaded into the lower center channel. The cell‐laden Matrigel was incubated for 1 h at 37°C for gelation. Following gelation, cell culture medium was introduced into the lower side channels to provide nutrients to the embedded cells and prevent Matrigel shrinkage. After 24 h (12 h for samples used in microglia polarization assays), the upper channel was coated with 50 µg/mL fibronectin for 30 min at 37°C, and HBMECs were seeded at a density of 7E7 cells/mL. After 1 h attachment period, the culture medium in the upper channel was replaced to remove unattached cells. The chips were then maintained at 37°C with 5% CO_2_ for 3 days, and the cell medium was refreshed twice a day. A mixed cell culture medium (1:1:1:1 of astrocyte medium: endothelial cell medium: pericyte medium: microglia medium) was used for all penta‐culture experiments.

HAs, GBM, microglia, pericyte, and HBMEC were labeled with CellTracker dyes (CellTracker Red CMTPX Dye, Thermo Scientific; CellTracker Green CMFDA Dye, Thermo Scientific; CellTracker Deep Red Dye, Thermo Scientific; CellTracker Deep Red Dye, Thermo Scientific; CellTracker Green CMFDA Dye, Thermo Scientific) and then incubated at 37°C for 30 min before seeding into the chips. Following the penta‐culture assembly, the lower and upper layers were imaged by adjusting z‐axis focal planes. Although partial spectral overlap exists among the CellTracker dyes, separating the imaging planes allowed clear discrimination of the two layers.

### 2.9 Immunocytochemistry

Samples were fixed for 15 min at room temperature (RT) using 2% paraformaldehyde (PFA; Bio‐solution, Suwon‐si, Gyeonggi‐do, Republic of Korea). To minimize contraction of HBMECs, astrocytes, and GBM cells, the PFA solution was pre‐warmed in a water bath prior to use. After fixation, samples were permeabilized with 0.1% Triton X‐100 (Sigma‐Aldrich) for 15 min at RT and blocked with 2% bovine serum albumin (BSA; Sigma‐Aldrich) for 1 h at RT.

Primary antibodies were introduced according to the specific experimental application (Table S1), followed by incubation for 3 h at RT and three washes with 1% BSA in PBS. Samples were then incubated with the corresponding fluorescence‐conjugated secondary antibodies for 1 h at RT (Table S1). Nuclear staining was performed using 1 µg/mL DAPI (D9542; Sigma‐Aldrich) for 5 min, followed by washing with DI water and PBS. Confocal imaging was conducted using an LSM 980 microscope (Carl Zeiss, Oberkochen, Germany) at the KAIST Analysis Center for Research Advancement (KARA), and image processing was performed using ZEN 3.1 blue edition.

### 2.10 Permeability Measurement

After 4 days of BBB/BBTB culture, culture medium supplemented with 4 kDa FITC‐dextran (500 µg mL^−^^1^; Sigma‐Aldrich) was perfused through the vascular channel at a flow rate of 16 µL min^−^^1^ using a syringe pump (Legato 210; KD Scientific). Concurrently, culture medium from the lower side channel was collected at 8 µL min^−^^1^ over a 1 h period. Fluorescence intensities of collected samples were quantified using a plate reader (Varioskan ALF; Thermo Fisher Scientific), and FITC‐dextran concentrations were determined using a standard calibration curve. The apparent permeability coefficient (*P_app_*) was calculated according to the following equation:

$$\;P_{app}=\frac{V}{A}\;\cdot \frac{dC/dt}{\Delta C}$$

where V is the volume of the collected sample, A is the surface area of the endothelial barrier, dC/dt is the rate of concentration change in the abluminal compartment over time, and ΔC is the concentration gradient across the barrier.

### 2.11 Transendothelial Electrical Resistance (TEER) Measurement

TEER was measured across the endothelial monolayer in each device using a custom‐built electrode system. The setup included Ag/AgCl wires (300 µm diameter, 30 mm length) integrated into a modified connector, and linked to an EVOM2 volt‐ohmmeter (World Precision Instruments, Sarasota, FL, USA), which applies a 10 µA alternating current at a frequency of 12.5 Hz. To minimize background measurement interference, the electrode wires were encased in Tygon tubing (0.8 mm inner diameter × 2.38 mm outer diameter; Duksan General Science, Republic of Korea) and PE‐60 tubing (0.68 mm ID × 1.18 mm OD; Musashi Engineering, Japan), both filled with culture medium. Each pair of Ag/AgCl electrodes was inserted into the inlet and outlet of the upper and lower channels, respectively. Resistance values were recorded after 1 min stabilization period. For each chip, ten readings were obtained and averaged. Final TEER values were derived by subtracting the resistance measured from blank models and multiplying the result by the endothelial‐covered surface area within the lower channel (0.015 cm^2^).

### 2.12 Real‐Time qRT‐PCR

HA, GBM cells, and ECs each at a density of 2E5 cells/mL, were seeded into a 6‐well plate and a transwell insert (Corning), respectively, and incubated at 37°C for 24 h. Subsequently, the inserts were transferred to the upper compartment. After 48 h, the culture medium and transwell insert were discarded, and cells were harvested at confluence using 0.25% Trypsin‐EDTA (GenDEPOT, TX, USA). Total RNA was extracted using Labozol reagent (CMRZ001, Cosmogenetech, Seoul, South Korea) following the manufacturer's instructions. Complementary DNA (cDNA) synthesis was performed by converting 1 µg of total RNA using the PrimeScript IV first strand cDNA Synthesis Mix (6215A, TAKARA, Kusatsu, Japan). RT‐qPCR was conducted on cDNA samples using SYBR Green (RT500M, Enzynomics, Daejeon, South Korea) to evaluate the expression levels of target genes. The gene expression data obtained from the experiments were normalized using the housekeeping gene (beta actin; *ACTB*). Primer sequences utilized in the experiments are detailed in Table S2.

For EC gene expression analysis in patient‐specific setups, ECs were seeded in 6‐well plates and HAs with GBM cells seeded in transwell insert (Corning) at a total amount of 1.5E5 cells per compartment at 37°C for 24 h. Subsequently, the inserts were transferred to the upper compartment. After 48 h, the culture medium and transwell insert were discarded, and ECs were lysed in 500 µ*L* of Trizol reagent (Invitrogen) for subsequent RNA extraction. Complementary DNA (cDNA) synthesis was performed by converting 1 µg of total RNA using iScript cDNA Synthesis kit (Bio‐Rad, Hercules, CA, USA). RT‐qPCR was conducted on cDNA samples using SYBR Green (SYBR Supermix; Bio‐RAD) to evaluate the expression levels of target genes. The gene expression data obtained from the experiments were normalized using the housekeeping gene (*GAPDH*). Primer sequences utilized in the experiments are detailed in Table S3.

### 2.13 TMZ and Bevacizumab Treatment and Efficacy Evaluation

TMZ (Sigma‐Aldrich) reconstituted in DMSO was added to culture media in concentration of 50 and 500 uM respectively. To investigate the chemotherapeutic specific response of cancer cells in GBM patients, co‐cultured cells were treated with or without 50 uM (for penta‐culture model: 50 µM only) and 500 µM TMZ for 72 h in a mixture of astrocyte media (AM) and GBM media in a 1:1 ratio. The mixed medium was filled into the medium channel of the device. Bevacizumab (BEV; Roche), a high–molecular‐weight monoclonal antibody with limited BBB permeability, was diluted in culture medium and administered at a single concentration of 200 µg/mL for 24 h respectively in a mixture of AM and GBM media in a 1:1 ratio [25]. The mixture medium was similarly delivered to the device.

Following TMZ and BEV treatment, to measure the cell viability of GBM cells per patient in response to TMZ treatment, HA and GBM cells in the lower channel of the device were stained with 0.5 µg/mL propidium iodide solution (PI; Biolegend, San Diego, CA, USA) in DPBS at room temperature in the dark for 1 h. Subsequently, to prevent nonspecific staining and remove excess PI dye, excess PI staining solution was carefully aspirated, and the cells were washed again with DPBS. The stained cells were imaged using a fluorescence microscope for PI detection in the Texas Red channel. The stained cell area was quantified using ImageJ, and the resulting cell area values were normalized against the average of the control group.

### 2.14 Culture Media Optimization

To evaluate primary GBM cell viability under different culture media conditions, a WST‐8 assay was performed using the WST‐8 Cell Counting Kit (QM1000, Biomax, Korea) (Figure S3). For this experiment, astrocytes and GBM cells cultured within the microfluidic chip were maintained for 3 days under six medium conditions: (1) GBM medium, (2) astrocyte medium, (3) endothelial medium, (4) a 1:1 mixture of astrocyte and endothelial media, (5) a 1:1 mixture of astrocyte and GBM media, and (6) a 1:1:1 mixture of astrocyte, GBM, and endothelial media. Each medium condition was refreshed daily during the 3 days incubation period. After 3 days, the culture supernatant from each chip was collected, and 100 µL of the conditioned medium was transferred into a 96‐well microplate. Subsequently, 10 µL of the WST‐8 reagent (10% of the total reaction volume) was added to each well according to the manufacturer's instructions. The plate was incubated for 1 h at 37°C in a 5% CO_2_ incubator to allow colorimetric reaction. Following incubation, the plate was gently shaken, and absorbance was measured at 450 nm using a colorimetric microplate reader. The measured optical density values were used to compare GBM cell viability across the six media conditions.

### 2.15 Reproducibility and Batch Control

All experiments were designed to minimize batch variability. TEER measurements were performed in two separate runs, while qPCR analyses were conducted simultaneously in a single batch. To further reduce potential variability, HAs, HBMECs, and HBVPs were used at passages 3–5, and the same lot of primary cells and Matrigel were used across all experiments. These measures were taken to ensure consistency in experimental conditions and strengthened the reproducibility of the observed trends despite the limited number of patient‐derived samples.

### 2.16 Statistics Analysis

All statistical analyses were performed using GraphPad Prism 10 (GraphPad Software, La Jolla, CA, USA). Comparisons between two groups were analyzed using Student’s t‐test, whereas comparisons among multiple groups were performed using one‐way ANOVA followed by Tukey’s multiple comparisons test. Statistical significance was determined based on the P‐values: P < 0.05(*), P < 0.01(**), P < 0.001(***) and P < 0.0001(****).

## 3 Results

### 3.1 Reconstruction of the Blood‐brain Tumor Barrier in GBM on Chip

The BBTB associated with GBM has been recapitulated using a double‐layered, compartmentalized microengineered chip model. The upper vascular channel was lined with a monolayer of HBMECs, while the lower tissue channel contained a 3D hydrogel‐based network of HAs and patient‐derived GBM cells (Figure 1B; Figure S1). These two layers were separated by a porous membrane, mimicking the vascular‐parenchymal interface. Continuous nutrient exchange and metabolic waste removal were maintained via side media channels integrated along the lower tissue channel (Figure 1C).

HBMECs cultured in the vascular channel formed a compact monolayer with continuous tight junctions (Figure 1D), while the co‐cultured HAs and GBM cells established a 3D cellular network within 72 h (Figure 1E). In the healthy BBB model, HBMECs displayed continuous tight junction (ZO‐1; zonula occludens‐1) network and prominent expression of glucose transporter 1 (GLUT1), consistent with intact and specialized endothelial phenotypes at the BBB (Figure 1D). Conversely, the BBTB model exhibited heterogeneous ZO‐1 organization, with junctions remaining continuous in some regions but appearing discontinuous or absent in others (Figure 1D). While overall GLUT1 immunofluorescence signals appeared comparable between conditions, subtle variations in GLUT1 localization were observed in the BBTB model, suggesting locally impaired endothelial metabolic specialization. Together, these structural features indicate a partially disrupted tight junction network and regionally compromised endothelial function driven by tumor‐derived influences.

Moreover, the BBTB model exhibited reduced barrier integrity, as indicated by increased permeability coefficient, compared to the healthy BBB model (Figure 1F). In contrast, TEER values did not show a statistically significant difference between the two conditions, despite the presence of discontinuous ZO‐1 localization along endothelial junctions (Figure 1G). This suggests that tight‐junction disorganization may increase molecular permeability before producing measurable changes in electrical resistance. This discrepancy reflects the fact that permeability reports on size‐dependent molecular transport, whereas TEER quantifies ionic conductance, capturing distinct aspects of barrier integrity. Furthermore, the presence of GBM cells did not significantly alter endothelial gene expression levels of junctional proteins, transporters, or receptors (Figure S4), indicating that our BBTB model recapitulates the structural preservation yet functional leakage often observed at the infiltrative margins of GBM. These findings align with previous reports indicating that the endothelial barrier remains stable at the GBM margins compared to the barrier in the highly vascularized tumor core [7, 26, 27].

### 3.2 Crosstalk between GBM and Astrocytes

Astrocytes located in the peritumoral regions of GBM, commonly referred to as tumor‐associated astrocytes, have been shown to promote the growth and progression of the tumor [28, 29], while GBM cells exploit these astrocytes to enhance their invasive potential and survival [30]. We co‐cultured astrocytes with GBM cells isolated from GBM patient tissues to investigate these bi‐directional interactions. Within the 3D microenvironment of our BBTB chip, both astrocytes and GBM cells exhibited branched and bushy shape, a physiological morphology observed in the brain‐resident glial cells (Figure 1E). Notably, the GBM cells were uniformly and randomly distributed among astrocytes, rather than forming spheroids (Figure 1E). This distribution closely replicates the heterogeneous microenvironment observed at the GBM margins, where infiltrative GBM cells intermix with resident astrocytes [31].

**FIGURE 2:**
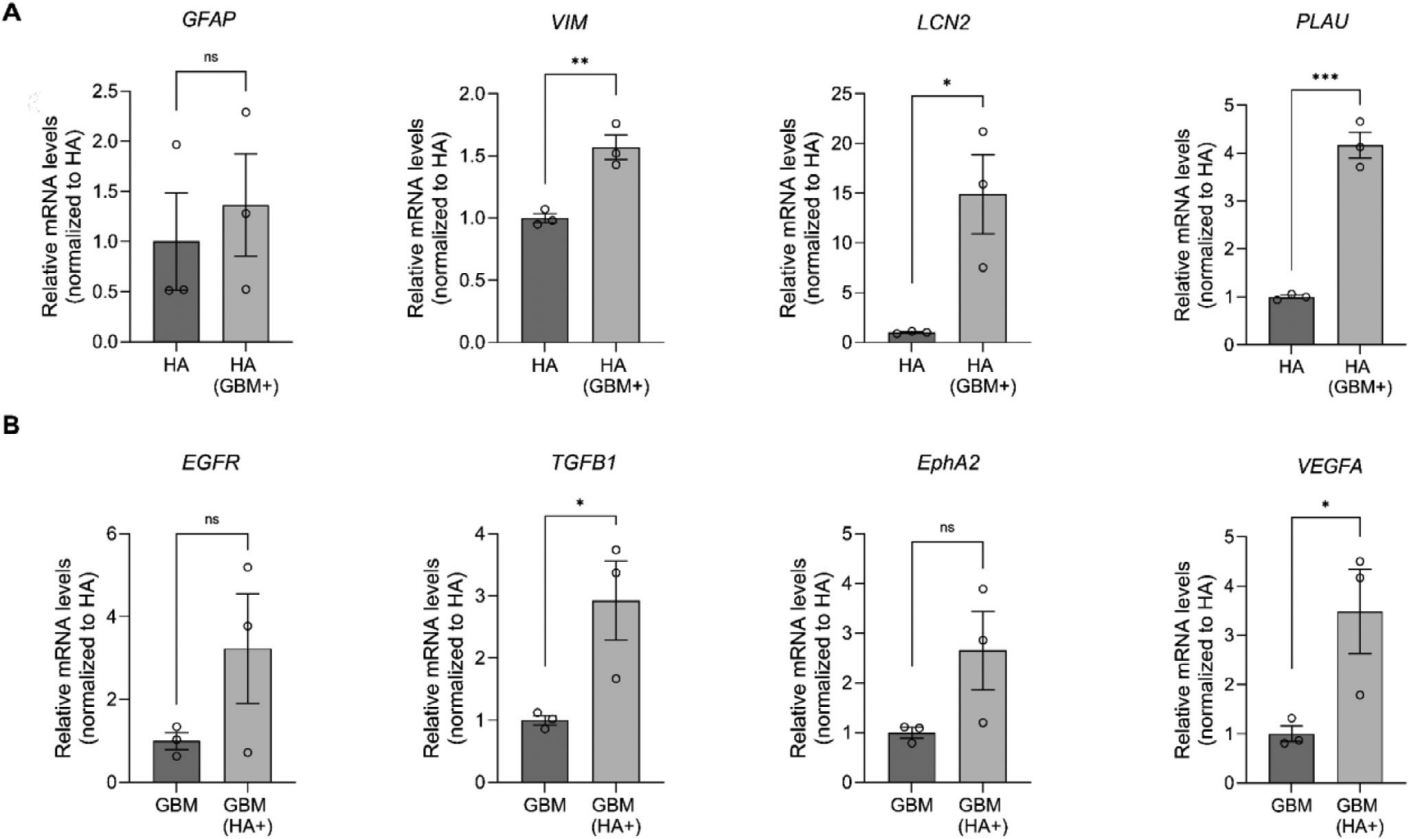
Interaction between HAs and GBMs. (A) Changes in gene expressions of HAs when co‐cultured with GBM cells and (B) changes in gene expression of GBM cells when co‐cultured with HAs, suggesting that HAs enhance tumor progression and invasiveness in the GBM environment. (n = 3 for each condition, *p < 0.05, **p < 0.01 and ***p < 0.001 by student's t‐test, error bars represent the standard error of the mean (SEM)).

The interactions between astrocytes and GBM cells resulted in the significant upregulation of gene expressions of reactive gliosis markers, such as vimentin (*VIM*) and lipocalin 2 (*LCN2*) in astrocytes (Figure 2A). Moreover, plasminogen activator, urokinase (*PLAU*), which encodes urokinase‐type plasminogen activator (uPA), was elevated in astrocytes in response to GBM contact (Figure 2A). uPA enhances the invasive capabilities of GBM cells via the uPA‐plasmin cascade, which increases the activation of metalloproteinases (MMPs) [30]. In parallel, GBM cells co‐cultured with astrocytes, showed the upregulated expression of transforming growth factor beta 1 (*TGF‐β*), and vascular endothelial growth factor A (*VEGFA*) (Figure 2B), indicating activation of pro‐invasive and pro‐angiogenic signaling pathways [28, 32]. In particular, *VEGFA* upregulation can also influence endothelial function by modulating vascular permeability [33]. Taken together, these results demonstrate a dynamic crosstalk between GBM cells and astrocytes, in which tumor‐derived signals activate astrocytes toward a reactive, tumor‐promoting phenotype, while astrocytes reciprocally enhance the invasive and pro‐angiogenic potential of GBM cells [30, 34].

### 3.3 Patient‐Specific Blood‐Brain Tumor Barrier Models

**FIGURE 3:**
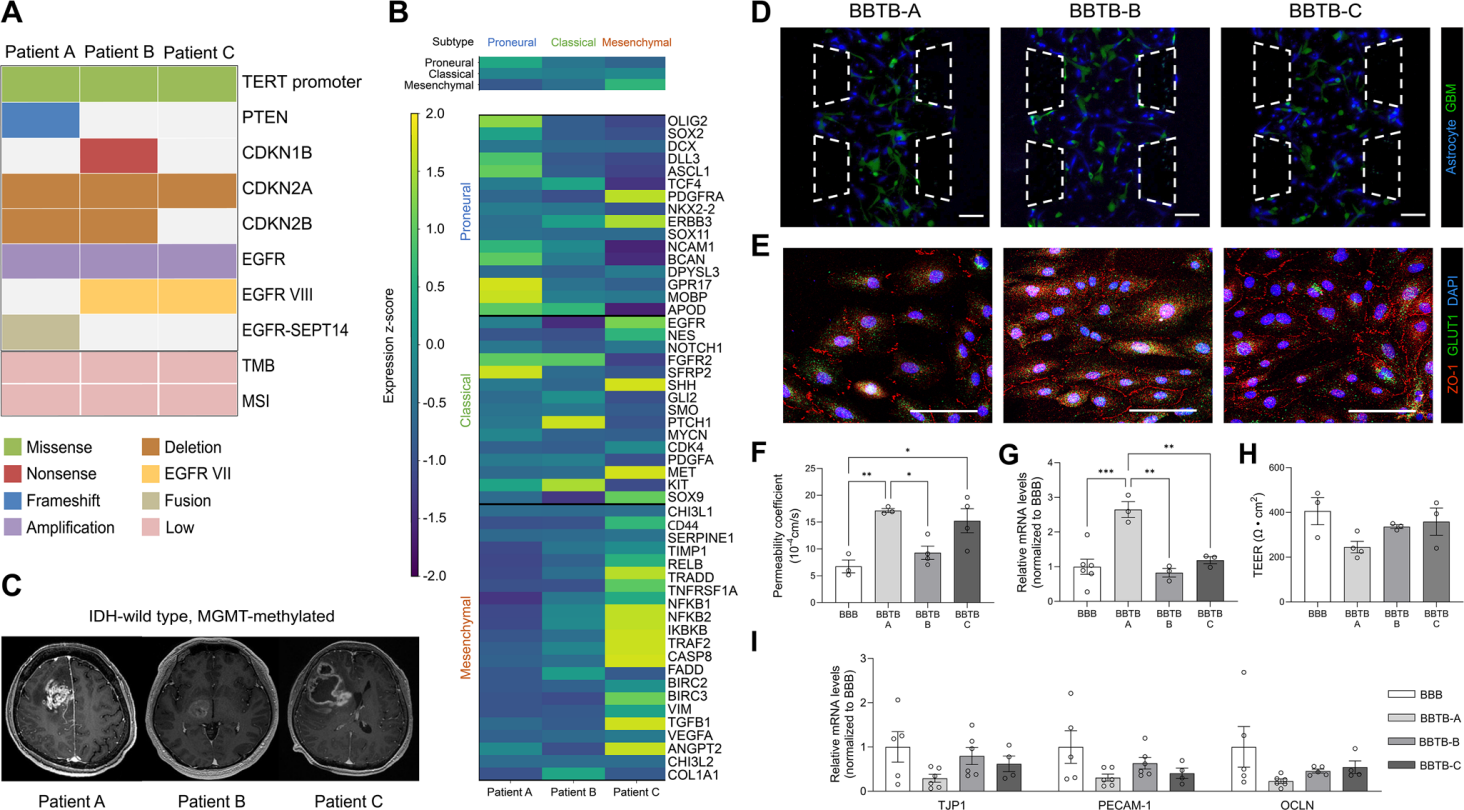
Establishment of the patient‐specific BBTB model. (A) Oncoplot summarizing small variants, copy number variations (CNVs), gene fusions, tumor mutational burden (TMB), and microsatellite instability (MSI) identified by panel sequencing. (B) Heatmap showing GBM subtype–associated transcriptional signatures for the three patient‐derived tumors. (C) Representative magnetic resonance images of GBM patients showing contrast‐enhancing lesions. (D) Co‐culture of HAs and GBM cells from different patients in our device (HAs, blue; patient‐derived GBM cells, green) (lower channel, All scale bars = 100 µm). (E) HBMEC monolayers formed in the vascular channel of each patient‐specific model (ZO‐1, red; GLUT1, green; DAPI, blue) (All scale bars = 100 µm). (F) Permeability coefficient values of BBB and patient‐specific BBTB models (Patients A‐C) (n = 3 for BBB and BBTB‐A, n = 4 for BBTB‐B and BBTB‐C, *p < 0.05 and **p < 0.01 by one‐way ANOVA with Tukey's post hoc test, error bars represent the standard error of the mean (SEM)). (G) Relative mRNA expression of efflux related gene ABCB1 in patient‐specific BBTB models, normalized to BBB controls (n = 6 for BBB, and n = 3 for BBTB‐A, BBTB‐B, and BBTB‐C, *p < 0.05 and **p < 0.01 by one‐way ANOVA with Tukey's post hoc test, error bars represent the standard error of the mean (SEM)). (H) TEER of BBB and patient‐specific BBTB models (Patients A‐C) (n = 3 for BBB and BBTB‐B, n = 4 for BBTB‐A and BBTB‐C, *p < 0.05 and **p < 0.01 by one‐way ANOVA with Tukey's post hoc test, error bars represent the standard error of the mean (SEM)). (I) Relative mRNA expression of endothelial junction‐related genes (*TJP1*, *PECAM‐1*, *OCLN*) in patient‐specific BBTB models, normalized to BBB controls (n = 5 for BBB, BBTB‐A, BBTB‐B, and n = 4 for BBTB‐C, *p < 0.05 and **p < 0.01 by one‐way ANOVA with Tukey's post hoc test, error bars represent the standard error of the mean (SEM)).

The BBTB represents a critical component of the GBM microenvironment, reflecting barrier function, the pathological state of tumor‐associated vasculature, and potential modulation of therapeutic efficacy [8, 35]. However, the extent of BBTB disruption remains highly heterogeneous among patients with GBM. To investigate inter‐patient variability, we established personalized BBTB models using GBM cells isolated from three patients with distinct molecular profiles (Figure 3A–E; Table S4).

Across the three patient‐derived BBTB models, we observed distinct patterns of barrier integrity that aligned with patient‐specific genomic backgrounds. BBTB‐A, derived from a proneural‐dominant tumor, exhibited more heterogeneous junctional organization, characterized by a discontinuous tight‐junction network and significantly increased permeability coefficients relative to the healthy BBB model (Figure 3E,F). Targeted NGS analysis revealed that, in addition to alterations common to all three tumors, Patient A uniquely harbored a PTEN mutation. Loss of PTEN function is known to destabilize endothelial junctions and increase vascular permeability, while EGFR structural variants can further potentiate aberrant signaling at the tumor–vascular interface [36]. Likewise, BBTB‐C, derived from a mesenchymal‐dominant GBM subtype frequently associated with vascular abnormality and enhanced BBB disruption, also exhibited increased permeability coefficients [37].

These combined oncogenic features provide a mechanistic explanation for the increased permeability coefficients observed in BBTB‐A and BBTB‐C compared with the healthy BBB model, while BBTB‐B exhibited permeability coefficient similar to those of the BBB model (Figure 3F). Interestingly, significant upregulation of the efflux transporter‐related gene *ABCB1* was observed only in BBTB‐A (Figure 3G), suggesting that, despite increased paracellular permeability, effective trans‐barrier drug accessibility in BBTB‐A may still be restricted by enhanced endothelial efflux activity.

In contrast to the permeability measurements, TEER values showed no significant differences among the BBB and all BBTB models (Figure 3H). Similarly, gene expression analysis of junction‐related markers did not demonstrate substantial changes across the models (Figure 3I). These findings suggest that the overall endothelial barrier architecture remained largely preserved across the models. One possible interpretation is that the increased permeability observed in BBTB‐A and BBTB‐C may arise from localized and heterogeneous junctional defects rather than complete barrier collapse. Such localized disruptions could permit enhanced molecular transport while minimally affecting overall ionic resistance, thereby resulting in elevated permeability despite relatively unchanged TEER values [38, 39].

Taken together, BBTB‐A and BBTB‐C exhibited more pathologically altered barrier phenotypes compared with the healthy BBB model, while BBTB‐B maintained barrier characteristics more comparable to those of the BBB model. Notably, all models were constructed using the same endothelial cells (HBMECs) and platform, suggesting that the observed differences in barrier function arise primarily from patient‐specific GBM cells. These findings highlight the strong modulatory influence of tumor‐derived factors on BBTB integrity and transport‐related barrier phenotypes.

### 3.4 Therapeutic Responses in Patient‐Specific BBTB Models

**FIGURE 4:**
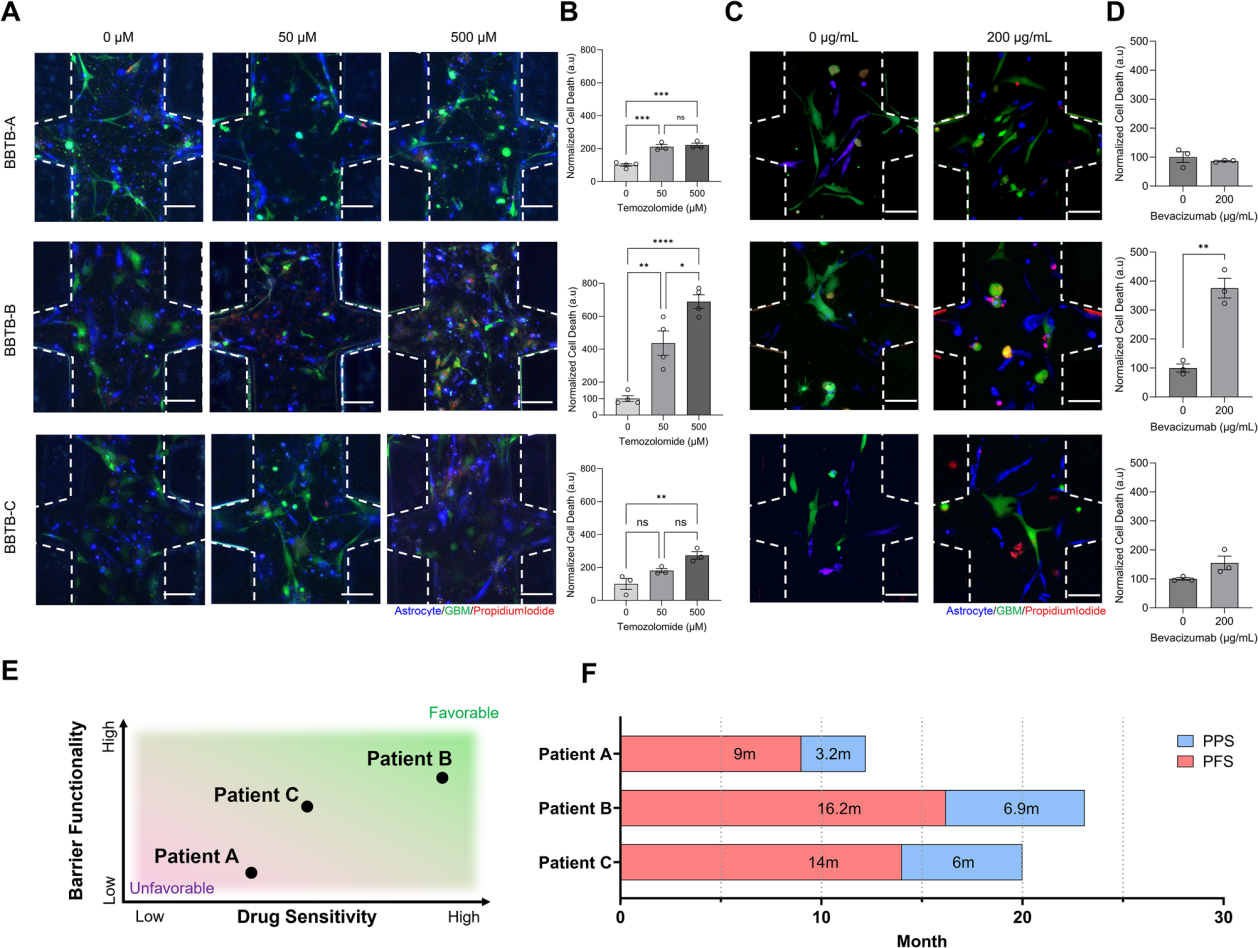
Chemotherapeutic responses in patient‐specific BBTB models. (A) Dose‐dependent viability of GBM cells in BBTB chips from three different patients (Patients A–C) treated with 0, 50, or 500 µM TMZ. (HAs, blue; patient‐derived GBM cells, green; PI (propidium iodide)‐stained cells, red) (All scale bars = 100 µm). (B) Quantification of PI fluorescence intensity normalized to control for each patient‐derived BBTB model under TMZ treatment (n ≥ 3 per group, *p < 0.05, **p <0.01 and ***p < 0.001 by one‐way ANOVA with Tukey's post hoc test. (C) Viability of GBM cells in BBTB‐A, B, and C treated with 0 or 200 µg/mL BEV. (HAs, blue; patient‐derived GBM cells, green; PI (propidium iodide)‐stained cells, red) (All scale bars = 100 µm). (D) Quantification of PI fluorescence intensity normalized to control for each patient‐derived BBTB model under BEV treatment (n ≥ 3 per group, *p < 0.05, **p < 0.01 and ***p < 0.001 by student's t‐test, error bars represent the standard error of the mean (SEM)). (E) Agreement between on‐chip findings and characteristics of the corresponding patient‐derived GBM, suggesting the model's ability to reflect individual tumor traits. (F) Swimmer plot illustrating progression‐free survival (PFS) and post‐progression survival (PPS) for each patient. PFS (red) represents the duration before disease progression, while PPS (blue) indicates the total survival period. Survival durations are shown in months (m).

All tumors were MGMT‐methylated and IDH‐wild‐type tumors, indicating their potential sensitivity to alkylating chemotherapy agents like TMZ (Figure 3C; Table S4). The efficacy of TMZ, the prime chemotherapy for GBM, has been known to be enhanced in MGMT promotor methylated tumors, by preventing tumor cells from repairing the drug‐induced DNA damage [21]. BEV, an anti‐angiogenic therapy, has been widely used for recurrent GBM after progression on standard TMZ‐based chemotherapy. However, the presence of diverse characteristics within GBM tumors can still lead to markedly different therapeutic outcomes even in patients with similar biomarker profiles. We first confirmed that TMZ treatment induces tumor cell death on three distinct patient‐specific models, using one clinically relevant low dose and one high dose commonly used in in vitro studies to account for TMZ instability [40, 41, 42, 43]. Differential drug sensitivities were observed across the models (Figure 4A; Figure S5). Among the three models, BBTB‐B exhibited the greatest sensitivity to TMZ, showing significant increases in tumor cell death at both 50 µM and 500 µM TMZ concentrations (Figure 4B). In contrast, BBTB‐A demonstrated a slight increase in cell death at 50 µM, but no further increase at 500 µM, suggesting a limited dose‐response relationship. BBTB‐C showed no significant change at 50 µM; however, a notable increase in cell death was observed between 50 µM and 500 µM (Figure S5), indicating a dose‐sensitive response at higher concentrations. Taken together, these results suggest a trend toward higher TMZ sensitivity in BBTB‐B, followed by BBTB‐C and BBTB‐A. These findings highlight robust patient‐specific variability in TMZ efficacy despite similar MGMT‐methylated status. Interestingly, MGMT‐unmethylated patient model (BBTB‐D), which is clinically known to exhibit strong resistance to TMZ, showed no significant increase in tumor cell death across all tested TMZ concentrations (Figure S6) [44]. This observation reflects commonly reported clinical trends, in which MGMT‐unmethylated GBM demonstrates markedly reduced sensitivity to alkylating therapy.

We next evaluated BEV, a high–molecular‐weight and low‐lipophilicity antibody with limited BBB permeability. BBTB‐A showed no detectable therapeutic effect. BBTB‐B displayed a statistically significant response, while BBTB‐C exhibited a mild but non‐significant trend toward increased cell death (Figure 4C,D). Importantly, our platform captured the two major phases of GBM therapy: first‐line TMZ therapy during the initial disease course and BEV treatment administered upon tumor progression (Table S5). The patient‐specific BBTB models revealed distinct relationships between vascular barrier function and chemotherapeutic response (Figures 3E–G and 4A–D). BBTB‐A demonstrated pronounced permeability and high TMZ penetration but minimal TMZ‐ and BEV‐ induced cytotoxicity, suggesting intrinsic tumor resistance despite vascular disruption. In contrast, BBTB‐B exhibited a relatively intact barrier yet exhibited strong responsiveness to both TMZ and BEV, suggesting inherent tumor sensitivity that was largely independent of vascular permeability. These findings suggest that increased barrier dysfunction alone does not necessarily translate into improved therapeutic outcomes, as aggressive GBM phenotypes may simultaneously exhibit altered vascular barrier states and reduced intrinsic drug sensitivity. Accordingly, the combined assessment of barrier‐related phenotypes and intrinsic drug sensitivity may serve as a more comprehensive framework for characterizing patient‐specific heterogeneity in GBM.

These sequential barrier‐related phenotypes and drug responses observed in the patient‐specific BBTB models closely matched the clinical treatment courses observed in the corresponding patients (Figure 4E,F). Patient A, whose BBTB‐A model showed the most altered barrier phenotype together with reduced response to TMZ and BEV, experienced the shortest progression‐free survival (PFS) under TMZ therapy and post‐progression survival (PPS) under BEV therapy. In contrast, patient B, whose tumor model‐maintained barrier properties more comparable to those of the healthy BBB and exhibited the strongest responses to both TMZ and BEV, showed the longest PFS followed by the greatest PPS prolongation after transitioning to BEV. Patient C demonstrated intermediate barrier‐related and drug‐response phenotypes, corresponding to intermediate clinical outcomes.

### 3.5 Stromal and Immune Modulation of Drug Response

**FIGURE 5:**
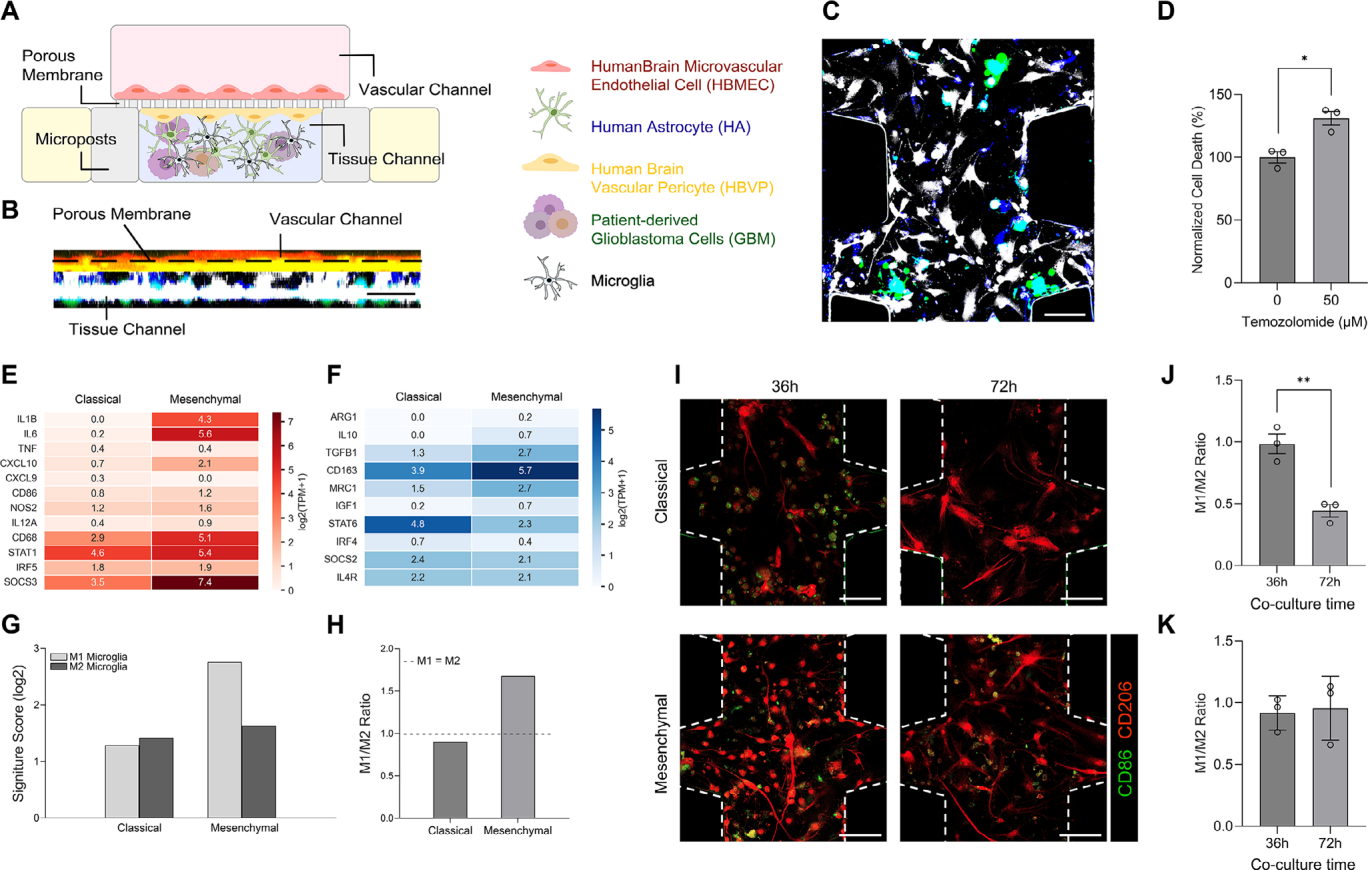
Drug response and microglial polarization in a stromal‐ and immune‐enhanced BBTB model. (A) Schematic representation of the penta‐culture BBTB within our device. (B) Side‐view z‐stack image of the stromal‐ and immune‐integrated BBTB model, showing the vertical spatial organization of HBMECs (red), human brain vascular pericytes (yellow), human astrocytes (blue), patient‐derived GBM cells (green), and microglia (white) (Scale bar = 100 µm). (C) 3D network of parenchymal cells, illustrating co‐culture of human astrocytes (blue), microglia (white), and GBM cells (green) (All scale bars = 100 µm). (D) TMZ response in the penta‐culture BBTB model, showing quantification of PI fluorescence intensity normalized to the untreated control. (n = 3 per group, *p < 0.05, **p < 0.01, ***p < 0.001 by Student's t‐test; error bars represent SEM). (E) M1 microglia marker expression in classical‐type and mesenchymal‐type GBM patients. (F) M2 microglia marker expression in classical‐type and mesenchymal‐type GBM patients. (G) Comparison of overall M1 and M2 microglia scores across classical and mesenchymal GBM patients. (H) M1/M2 ratio plotted according to microglia score, illustrating subtype‐specific immune polarization differences. (I) Immunofluorescence images of microglial polarization at 36 h and 72 h in classical, mesenchymal BBTB models, showing M1 (CD86, green) and M2 (CD206, red) phenotypes (All scale bars = 100 µm). (J) Quantification of M1/M2 ratio over time (36 h vs. 72 h) in the classical BBTB model (n = 3 per group, *p < 0.05, **p < 0.01, ***p < 0.001 by Student's t‐test; error bars represent SEM). (K) Quantification of M1/M2 ratio over time (36 h vs. 72 h) in the mesenchymal BBTB model (n = 3 per group, *p < 0.05, **p < 0.01, ***p < 0.001 by Student's t‐test; error bars represent SEM).

To further enhance the physiological relevance of our BBTB model and examine how stromal and immune components regulate therapeutic response, we incorporated HBVPs and microglia into the platform (Figure 5A). HBVPs were cultured on the porous membrane in the lower tissue channel, positioned opposite of the endothelial monolayer, while microglia were embedded within the 3D hydrogel containing HAs and GBM cells (Figure 5A). Fluorescence imaging confirmed successful establishment of a penta‐culture BBTB model, where endothelial‐pericyte layers are formed with 3D networks of HAs, GBM cells, and microglia (Figure 5B,C).

We then examined how the addition of HBVPs and microglia altered chemotherapeutic response. Compared with the BBTB model consisting of HBMEC, HAs, and GBM cells, the penta‐culture model exhibited smaller increase in TMZ‐induced tumor cell death at the same TMZ concentration (Figures 4B and 5D). This attenuation in TMZ efficacy suggests that stromal and immune components provide an additional layer of chemoresistance. These results are consistent with previous studies demonstrating that pericytes can enhance GBM resistance to TMZ, while M2‐polarized microglia promote GBM growth and survival [45, 46].

Microglial polarization is a critical determinant of GBM progression and treatment response, underscoring the importance of assessing whether patient‐specific immune states are recapitulated in our model. Bulk tumor RNA‐seq analysis from a matched GBM cohort revealed that Patient B (MGMT‐methylated, classical subtype) showed relatively balanced M1 and M2 gene signatures, whereas Patient D (MGMT‐unmethylated, mesenchymal subtype) exhibited elevated M1‐associated signatures (Figure 5E–H). To explore these trends in our platform, we assessed CD86 (M1) and CD206 (M2) expression in microglia at 36 and 72 h after co‐culture within our penta‐culture BBTB model (Figure 5I). In the classical GBM model, CD86+ and CD206+ microglia were present at comparable levels at 36 h, followed by a relative decline in M1 polarization at 72 h (Figure 5J). In contrast, the mesenchymal GBM model maintained a higher proportion of CD86+ microglia across both time points (Figure 5K). These subtype‐dependent differences in microglial dynamics reflect the clinical transcriptomic signatures and support the ability of our platform to capture individualized immune microenvironmental states.

## 4 Discussion

In this study, we developed a microengineered BBTB‐on‐a‐chip model that reconstructs key features of the GBM margin, where the BBTB serves as the critical checkpoint for therapeutic agents [47, 48]. Unlike many tumor‐on‐chip systems that primarily model the highly permeable vasculature of the tumor core, our platform captures the partially intact but spatially disrupted BBTB characteristic of the infiltrative margin, enabling functional assessment of drug accessibility in a clinically relevant microenvironment. To replicate the multifaceted biology of this perivascular niche, our model incorporates not only brain endothelial cells and astrocytes but also patient‐derived GBM cells, pericytes, and microglia. This configuration facilitates the formation of a tight‐junction‐forming endothelial monolayer and establishes a 3D perivascular niche, thereby enabling evaluation of GBM contributions to barrier stability, GBM‐astrocyte interactions, and immune components that regulate therapeutic resistance.

The margin zone of GBM, which lies between healthy brain tissue and the GBM core, is extremely challenging for identification and surgical removal. Despite its lower density of tumor cells compared to the GBM core, the infiltrative characteristics of GBM cells contribute to recurrence and progression of GBM [49, 50]. Moreover, the margin acts as a critical hurdle for drug delivery in tumors due to its less disrupted BBTB [51] and a significant diffusion barrier [5]. The interaction between normal astrocytes and GBM cells induces reactive gliosis, which is strongly associated with the diffusion properties of GBM and tumor progression [28, 52]. We have demonstrated increased reactive gliosis and invasive capacity of GBM cells through their interactions by examining the expression levels of the related genes.

In the GBM margin microenvironment, the integrity of BBTB is weakened, and such physical disruption can influence drug permeability. This addresses that drug efficacy is not solely determined by the intrinsic sensitivity of tumor cells, but also by the accessibility of therapeutic agents across the vascular barrier. BBTB disruption is neither uniform nor complete where certain regions may display leaky vasculature, others retaining intact tight junctions. In addition to this spatial heterogeneity, inter‐patient heterogeneity further contributes to diverse barrier integrity, with some patients showing highly permeable BBTB and others exhibiting relatively preserved barriers. As drug permeability varies among patients, patient‐specific assessment of barrier integrity may enable personalized treatment planning [43]. We evaluated the patient‐specific barrier integrity by measuring permeability coefficients and TEER across the patient BBTB models. Our results confirmed substantial heterogeneity in barrier‐related phenotypes among patients. Notably, one case exhibited a profoundly disrupted BBTB among the three patient‐derived models. This was further supported by the increased permeability and upregulation of efflux transporter‐related gene *ABCB1*. Although shear stress can influence baseline barrier properties, prior studies have shown that simplified static systems sufficiently recapitulate GBM‐induced barrier disruption, and our patient‐derived models consistently reproduced these pathological trajectories [53, 54]. Dynamic flow will be incorporated in future versions to enhance physiological relevance, but the current platform already provides a robust and modular system for dissecting patient‐specific vascular and immune mechanisms at the GBM margin.

Together with assessing barrier‐related phenotypes, we also examined patient‐specific drug responsiveness in our models using TMZ, a low molecular weight chemotherapeutic, and BEV, a high molecular weight monoclonal antibody. Although all three patients were diagnosed with IDH‐wildtype GBM and exhibited MGMT promoter methylation, our model revealed considerable differences in TMZ and BEV responsiveness among their respective BBTB models. In particular, therapeutic responses to small‐molecule agents such as TMZ may be affected by not only by BBTB permeability, but also by efflux‐related transport activity and intrinsic tumor sensitivity, which may reflect GBM aggressiveness and pathological vascular dysfunction. These findings emphasize that genomic markers alone may be insufficient to predict therapeutic outcomes. Expanding the use of patient‐specific models to evaluate responses to a broader range of therapeutic agents could provide actionable insights for personalized therapy.

Further incorporation of HBVPs and microglia into the BBTB model enabled us to investigate stromal‐ and immune‐mediated contribution to chemoresistance. Pericytes have been shown to promote DNA damage repair in GBM through CCL5–CCR5 signaling, while the polarization state of microglia strongly influences tumor invasion and therapeutic response [45, 55]. Consistent with these reported roles, the expanded penta‐culture model exhibited reduced TMZ responsiveness and demonstrated subtype‐dependent microglial polarization patterns that mirrored the transcriptomic signatures observed in clinical datasets. Importantly, the ability of our platform to preserve patient‐specific immune phenotypes underscores its utility for examining personalized immune contributions to drug resistance and for dissecting drug‐barrier‐immune interactions that are not accessible in conventional in vitro GBM models.

Our platform delivers results within two weeks of post‐surgery, offering an opportunity to guide personalized treatment early in the clinical timeline. While standard clinical markers are often insufficient to predict therapeutic responses and patient prognosis, simultaneous assessment of barrier‐related phenotypes and drug sensitivity in our functional model may provide a more comprehensive evaluation of GBM pathological states, thereby potentially enhancing clinical decision‐making. There is ample room for advancement in further characterizing the pathological features by incorporating analyses such as protein expression and cytokine profiling. Moreover, further incorporation of resistance‐enriched or post‐therapy cell states would allow evaluation of true post‐treatment biology and improve the translational relevance of the system. Altogether, we believe our model offers a valuable platform for exploring the GBM margin and the BBTB. This approach may further support the development of personalized GBM therapeutic strategies.

## Author Contributions

M.S.R., S.J.C., and S.I.A. wrote the manuscript. M.S.R., J.H.A., G.E.L., N.Y.K., S.J.H., S.J.C., and J.W.J. performed experiments. M.S.R., J.H.A., G.E.L., J.W.K., and S.J.C. analyzed data. G.E.L., S.H.H., and M.S.R. performed RT‐qPCR experiments. M.S.R., J.W.J., and S.J.C. performed immunocytochemistry. M.S.R., J.W.J., N.Y.K., and S.J.C. fabricated microfluidic devices. Y.J.K., J.M.S., and J.M.P. performed GBM cell isolation and culture. J.H.A., S.I.A., Y.J.K., and J.J.L. conceived, initiated, and supervised the overall project.

## Funding

National Research Foundation of Korea (NRF) grant funded by the Korea government (MSIT) (RS‐2026‐25487930 to S.I.A.; RS‐2024‐00357185 to J.J.L.; RS‐2024‐00350442 and RS‐2026‐25497630 to J.H.A.); Korea‐US Collaborative Research Fund (KUCRF), funded by the Ministry of Science and ICT and Ministry of Health & Welfare, Republic of Korea (RS‐2024‐00468873 to S.I.A.); the Korean fund for Regenerative Medicine grant funded by the Ministry of Science and ICT, and the Ministry of Health & Welfare (22A0106L1 to Y.J.K.); the Korea Health Technology R&D Project through the Korea Health Industry Development Institute, funded by the Ministry of Health & Welfare (RS‐2024‐00406054 to J.H.A.); a grant from the Industry‐Academic Cooperation Foundation of CHA University (Grant No. CHA‐202501360001).

## Ethics Statement

The use of patient tissues was approved by the Institutional Review Board (IRB No. CHAMC2021‐01‐024‐001) at CHA Bundang Medical Center, Republic of Korea, with written informed consent obtained from all patients.

## Conflicts of Interest

The authors declare no conflicts of interest.

## Supporting information




**Supporting File**: smll74191‐sup‐0001‐SuppMat.docx.

## Data Availability

All relevant data supporting the findings of this study are available within the paper and supplementary information files, and other data that support this study are available from the corresponding author upon reasonable request.
