# Supplementary material for: Human Blood‐Brain Tumor Barrier on a Chip to Investigate Personalized Treatment for Glioblastoma Patients
Source: Small. 2026 Jun 27;22(49):e06712. doi: 10.1002/smll.202506712 (PMC13533249; doi:10.1002/smll.202506712)
Supplement: Supplementary file 1 [file SMLL-22-e06712-s001.docx]

Supporting Information

**Human blood-brain tumor barrier on a chip to investigate personalized treatment for**

**glioblastoma patients**

Minsu Ryoo^1^†, Gaeun Lee^2^†, Jinwoo Jung^1^, Sujin Cho^3^, Sharon Jeeho Ham^4^, Nayeong Kang^1^, Hyeongjin Ahn^2^, Yu Jin Kim^5^, JeongMin Sim^5^, Jeongman Park^5^, Juwon Kim^5^, Sohyun Hwang^6^, Jihwan Yoo^7^ Youn-Jung Kang^9^*, Jaejoon Lim^5,8^*, Jungho Ahn^2,4^*, and Song Ih Ahn^1^*

†These authors contributed equally to this work and share first authorship.

**Affiliations:**

^1^ Department of Mechanical Engineering, Korea Advanced Institute of Science and Technology, Daejeon, Republic of Korea (M.S.R., J.W.J., N.K., S.I.A.)

^2^ Department of MetaBioHealth, Sungkyunkwan University, Suwon, Republic of Korea (G.E.L.,S.H.J., H.A., J.H.A.)

^3^ School of Mechanical Engineering, Pusan National University, Busan, Republic of Korea (S.J.C.)

^4^ Department of Biophysics, Sungkyunkwan University, Suwon, Republic of Korea (S.J.H., J.H.A.)

^5^ Department of Biomedical Science, School of Life Science, CHA University, Seongnam, Republic of Korea (J.M.S., J.M.P., J.W.K., J.J.L.)

^6^ Department of Pathology, Bundang CHA Medical Center, CHA University, Seongnam, Republic of Korea (S.H.H.)

^7^ Department of Neurosurgery, Brain Tumor Center, Gangnam Severance hospital, Seoul, Republic of Korea (J.H.Y)

^8^ Department of Neurosurgery, Bundang CHA Medical Center, CHA University, Seongnam, Republic of Korea (J.J.L.)

^9^ Department of Biochemistry, Research Institute for Basic Medical Science, School of Medicine, CHA University, Seongnam, Republic of Korea (Y.J.K.)

Corresponding authors:

Song Ih Ahn, PhD, Department of Mechanical Engineering, Korea Advanced Institute of Science and Technology (KAIST), 291 Daehak-ro, Yuseong-gu, Daejeon 34141, Republic of Korea (Tel : +82-42-350- 3015, [siahn@kaist.ac.kr](mailto:siahn@kaist.ac.kr));

Jungho Ahn, MD, PhD, Department of Biophysics, Sungkyunkwan University, 2066, Seobu-ro, Jangan-gu, Suwon-si, Gyeonggi-do, 16419, Republic of Korea (Tel: +82-31-299-4797, [jhahn513@skku.edu](mailto:jhahn513@skku.edu));

Jaejoon Lim, MD, PhD, Department of Biomedical Science, School of Life Science;Department of Neurosurgery, Bundang CHA Medical Center, 59, Yatap-ro, Bundang-gu, Seongnam-si, Gyeonggi-do, 13496, Republic of Korea (Tel: +82-31-780-5688, [coolppeng@chamc.co.kr](mailto:coolppeng@chamc.co.kr));

Youn-Jung Kang, PhD, Department of Biochemistry, Research Institute for Basic Medical Science, School of Medicine, CHA University - Bundang Campus, 335, Pangyo-ro, Bundang-gu, Seongnam-si, Gyeonggi-do, 13488, Republic of Korea (Tel: +82- 31-881-7174, [yjkang@cha.ac.kr](mailto:yjkang@cha.ac.kr));

**Supplementary figure S1. A photo of the fabricated device.** The upper channel of the device made of PDMS is marked with red ink and the lower center channel is marked with blue ink (right). A magnified view of the lower channels (left) shows cells in hydrogel in the lower center channel bordered by microposts and culture medium in the lower side channel (scale bar = 100 µm).

**Supplementary figure S2. Bright field images of glioma cells from GBM patients utilized in this study.**

**Supplementary figure S3.** Cell viability was assessed after 3 days of culture in six media formulations: astrocyte medium (AM), GBM medium (GM), endothelial medium (EM), a 1:1 mixture of AM and GM (AGM), a 1:1 mixture of AM and EM (AEM), and a 1:1:1 mixture of AM, GM, and EM (ACEM). No significant differences were observed between GM and the mixed-media conditions, confirming that the mixed astrocyte–endothelial medium supports stable GBM viability.

**Supplementary figure S4. Comparison of relative mRNA levels in endothelial cells under mono- and co-culture conditions.** (n=3 for each condition, *p<0.05, **p<0.01 and ***p<0.001 by student’s t-test).

**Supplementary figure S5. Normalized cell death rates of patient-derived GBM cells following TMZ treatment in three BBTB models. (A)** Cell death response at 50 µM TMZ(n = 3, *p<0.05, **p<0.01 and ***p<0.001 by one-way ANOVA with Tukey's post hoc test, error bars represent the standard error of the mean (SEM)). **(B)** Cell death response at 500 µM TMZ. Values are normalized to the respective baseline response at 0 µM TMZ for each model. (*p<0.05 and ***p<0.001 by one-way ANOVA with Tukey's post hoc test, error bars represent the standard error of the mean (SEM)) **(C)** Comparison of TMZ drug sensitivity across patient-derived BBTB models. Drug sensitivity was quantified as the difference in normalized cell death between 500 µM and 50 µM TMZ treatment (n = 3, *p<0.05, **p<0.01 and ***p<0.001 by one-way ANOVA with Tukey's post hoc test, error bars represent the standard error of the mean (SEM)).

**Supplementary figure S6. Construction and drug testing of the MGMT-unmethylated patient-derived BBTB model.** **(A)** Magnetic resonance images (MRI) scan of the patient highlighting contrast-enhancing GBM lesions. **(B)** Oncoplot summarizing small variants, copy number variations (CNVs), gene fusions, TMB and MSI score identified by panel sequencing. **(C)** Heatmap showing GBM subtype–associated transcriptional signatures of patient-derived tumor. **(D)** Co-culture of astrocytes and GBM cells of MGMT unmethylated patient in our device (lower channel, HAs, blue; GBM cells, green, scale bars = 100 µm). **(E)** TEER comparison between the healthy BBB and the MGMT unmethylated BBTB-D model (n = 3, *p<0.05, **p<0.01 and ***p<0.001 by one-way ANOVA with Tukey's post hoc test, error bars represent the standard error of the mean (SEM)). **(F)** Relative mRNA expression of endothelial junction-related genes (*TJP1*, *PECAM-1*, *OCLN*) in BBTB-D model, normalized to BBB controls. **(G)** Dose-dependent viability of GBM cells in BBTB-D chips after treatment with 0, 50, or 500 µM TMZ. (HAs, blue; patient-derived GBM cells, green; PI (propidium iodide)-stained cells, red) (All scale bars = 100 µm). **(H)** Quantification of PI fluorescence intensity normalized to control BBTB-D model under TMZ treatment (n = 3, *p<0.05, **p<0.01 and ***p<0.001 by one-way ANOVA with Tukey's post hoc test, error bars represent the standard error of the mean (SEM)).

| Experiment | Antibody | Sequence |
| --- | --- | --- |
| **Endothelial junction, cell proliferation and spatial distribution** | Primary | Rabbit anti–Ki-67 (SAB5700770; 1:100; Sigma-Aldrich),  Mouse anti–ZO-1 (Alexa Fluor 594 conjugated; 339194; 1:100; Invitrogen), Mouse anti-GFAP (Alexa Fluor 488 conjugated; 53-9892-82; 1:100; eBioscience) |
|  | Secondary | Goat anti-rabbit Alexa Fluor 555 (A21428; 1:200; Invitrogen) |
| **Endothelial junction and glucose transporter assessment** | Primary | Rabbit anti–GLUT1 (ZRB071401; 1:100; Sigma-Aldrich), Mouse anti–ZO-1 (Alexa Fluor 594 conjugated; 339194; 1:100; Invitrogen) |
|  | Secondary | Chicken anti-rabbit Alexa Fluor 647 (A21443; 1:200; Invitrogen) |
| **Microglial polarization analysis** | Primary | Rabbit anti-CD206 (mannose receptor; ab64693; 1:900; Abcam), Mouse anti-CD86 (ab220188; 1:200; Abcam) |
|  | Secondary | Chicken anti-rabbit Alexa Fluor 647 (A21443; 1:200; Invitrogen), Goat anti-mouse Alexa Fluor 488 (A21121; 1:200; Invitrogen) |

**Supplementary Table S1. Primary and secondary antibodies used in immunocytochemistry**

| Gene | Direction | Sequence |
| --- | --- | --- |
| *ACTB* | Forward | CATGTACGTTGCTATCCAGGC |
|  | Reverse | GCCTTAATGTCACGCACGAT |
| *GFAP* | Forward | CTGGAGAGGAAGATTGAGTCGC |
|  | Reverse | ACGTCAAGCTCCACATGGACCT |
| *VIM* | Forward | AGGCAAAGCAGGAGTCCACTGA |
|  | Reverse | ATCTGGCGTTCCAGGGACTCAT |
| *LCN2* | Forward | GTGAGCACCAACTACAACCAGC |
|  | Reverse | GTTCCGAAGTCAGCTCCTTGGT |
| *PLAU (uPA)* | Forward | CTGTCACCTACGTGTGTGGAG |
|  | Reverse | TGAGCGACCCAGGTAGACG |
| *EGFR* | Forward | AACACCCTGGTCTGGAAGTACG |
|  | Reverse | TCGTTGGACAGCCTTCAAGACC |
| *TGFB1* | Forward | AGAGTGTCTGCGGATACTTCC |
|  | Reverse | CCAACAGTGTAGGTCTTGGTG |
| *EphA2* | Forward | ACTGCCAGTGTCAGCATCAACC |
|  | Reverse | GTGACCTCGTACTTCCACACTC |
| *VEGFA* | Forward | AGGAGGGCAGAATCATCACGAA |
|  | Reverse | AGGGTCTCGATTGGATGGCA |
| *ZO-1* | Forward | GTCCAGAATCTCGGAAAAGTGCC |
|  | Reverse | CTTTCAGCGCACCATACCAACC |
| *PECAM1* | Forward | AAGTGGAGTCCAGCCGCATATC |
|  | Reverse | ATGGAGCAGGACAGGTTCAGTC |
| *GLUT1* | Forward | TTGCAGGCTTCTCCAACTGGAC |
|  | Reverse | CAGAACCAGGAGCACAGTGAAG |
| *LRP1* | Forward | CAACGGCATCTCAGTGGACTAC |
|  | Reverse | TGTTGCTGGACAGAACCACCTC |
| *OCLN* | Forward | ATGGCAAAGTGAATGACAAGCGG |
|  | Reverse | CTGTAACGAGGCTGCCTGAAGT |
| *ABCB1* | Forward | GGGAGCTTAACACCCGACTTA |
|  | Reverse | GCCAAAATCACAAGGGTTAGCTT |

**Supplementary Table S2. Primer sequences used for qRT-PCR**

| Gene | Direction | Sequence |
| --- | --- | --- |
| *GAPDH* | Forward | ACAACTTTGGTATCGTGGAAGG |
|  | Reverse | GCCATCACGCCACAGTTTC |
| *TJP1* | Forward | CAACATACAGTGACGCTTCACA |
|  | Reverse | CACTATTGACGTTTCCCCACTC |
| *PECAM-1* | Forward | AACAGTGTTGACATGAAGAGCC |
|  | Reverse | TGTAAAACAGCACGTCATCCTT |
| *OCLN (Occludin)* | Forward | ACAAGCGGTTTTATCCAGAGTC |
|  | Reverse | GTCATCCACAGGCGAAGTTAAT |

**Supplementary Table S3. Primer sequences used for qRT-PCR in patient-specific models**

|  | Age/ Sex | Diagnosis | IDH mutation | *ATRX* | *H3K27M* | *GFAP* | *Ki-67* | *MGMT* |
| --- | --- | --- | --- | --- | --- | --- | --- | --- |
| PT#A | 62/ F | Glioblastoma,  newly diagnosed | Wild-type | No Loss | Negative | Positive | 42% | Methylation |
| PT#B | 41/ M | Glioblastoma,  newly diagnosed | Wild-type | No Loss | Negative | Positive | 35% | Methylation |
| PT#C | 44/ M | Glioblastoma,  newly diagnosed | Wild-type | No Loss | Negative | Positive | 30% | Methylation |

**Supplementary Table S4. Clinical and molecular characteristics of GBM patients**

| Patient | Drug | Cycles |
| --- | --- | --- |
| Patient A | TMS | Standard treatment complete |
|  | BEV | 2 cycle |
| Patient B | TMZ | Standard treatment complete |
|  | BEV | 8 cycle |
| Patient C | TMZ | Standard treatment complete |
|  | BEV | 5cycle |
| Patient MGMT unmethylated | TMZ | Standard treatment on-going |
|  | BEV | (-) |

**Supplementary Table S5. Summary of Treatment Duration per Patient and Drug**
